# Pathological functional impairment: Neuropsychological correlates of the shared variance between everyday functioning and brain volumetrics

**DOI:** 10.3389/fnagi.2022.952145

**Published:** 2022-12-23

**Authors:** Robert P. Fellows, Katherine J. Bangen, Lisa V. Graves, Lisa Delano-Wood, Mark W. Bondi

**Affiliations:** ^1^Psychology Service, VA San Diego Healthcare System, San Diego, CA, United States; ^2^Department of Psychiatry, University of California, San Diego, San Diego, CA, United States; ^3^Research Service, VA San Diego Healthcare System, San Diego, CA, United States; ^4^Department of Psychology, California State University, San Marcos, CA, United States

**Keywords:** instrumental activities of daily living, cognition, white matter hyperintensities, mild cognitive impairment, hippocampus, memory, Alzheimer’s disease, attention

## Abstract

**Objective:**

Given that several non-cognitive factors can contribute to difficulties with everyday functioning, examining the extent to which cognition is associated with brain-related changes in everyday functioning is critical to accurate characterization of cognitive disorders. In this study, we examined neuropsychological correlates of the shared variance between everyday functioning and pathological indicators of cognitive aging using MRI brain volumetrics.

**Participants and methods:**

Participants were 600 adults aged 55 and older without dementia [432 cognitively normal; 168 mild cognitive impairment (MCI)] from the National Alzheimer’s Coordinating Center cohort who underwent neuropsychological testing, informant-rated everyday functioning, and brain MRI scanning at baseline. The shared variance between everyday functioning and brain volumetrics (i.e., hippocampal volume, white matter hyperintensity volume) was extracted using the predicted value from multiple regression. The shared variance was used as an indicator of pathological everyday functional impairment. The residual variance from the regression analysis was used to examine functional reserve.

**Results:**

Larger white matter hyperintensity volumes (*p* = 0.002) and smaller hippocampal volumes (*p* < 0.001) were significantly correlated with worse informant-rated everyday functioning. Among individuals with MCI, worse performances on delayed recall (*p* = 0.013) and category fluency (*p* = 0.012) were significantly correlated with pathological functional impairment in multiple regression analysis. In the cognitively normal group, only worse auditory working memory (i.e., digit span backward; *p* = 0.025) significantly correlated with pathological functioning. Functional reserve was inversely related to anxiety (*p* < 0.001) in the MCI group and was associated with depressive symptoms (*p* = 0.003) and apathy (*p* < 0.001) in the cognitively normal group.

**Conclusion:**

Subtle brain-related everyday functioning difficulties are evident in MCI and track with expected preclinical Alzheimer’s disease cognitive phenotypes in this largely amnestic sample. Our findings indicate that functional changes occur early in the disease process and that interventions to target neuropsychiatric symptoms may help to bolster functional reserve in those at risk.

## Introduction

Older age in adulthood is associated with brain morphological changes (e.g., cortical atrophy, increased white matter disease) and declines in aspects of cognition (Salthouse, 2011; Zhao et al., 2019). The extent to which these factors relate to everyday functioning has important implications for identifying those at risk for pathological cognitive aging. Thresholds for diagnosing neurocognitive disorders rely, in part, on estimating the extent to which cognition contributes to functional ability. One of the primary distinctions between cognitive disorder classifications [e.g., cognitively normal, mild cognitive impairment (MCI), dementia] is the level of independence in instrumental activities of daily living (IADLs). However, several non-cognitive factors (e.g., physical functioning, psychological distress) can influence everyday functioning and complicate clinical and research determinations of brain-mediated functional disability (Vermeulen et al., 2011; Burton et al., 2018).

The term “pathological functional impairment” is used in the current study to represent the overlap between brain morphology consistent with known correlates of cognitive decline (i.e., white matter hyperintensity volume and hippocampal atrophy) and everyday functioning. This approach allows for functional difficulties related to brain volumetrics to be examined as both a continuous variable across the cognitive aging spectrum as well as within and between discrete diagnostic classifications (e.g., cognitively normal, MCI). The term “pathological functional impairment” is not intended to provide a static definition, but rather it is intended to represent a dynamic concept that can be applied to the study of disease in different populations and evolve and change.

In a systematic review of 20 studies examining correlates of everyday functioning in older adults, neuropsychological performance and brain morphology (hippocampal atrophy and white matter changes, in particular) were both unique and overlapping predictors of IADLs (Overdorp et al., 2016). These findings suggest a differential influence of structural brain changes on cognition and functional decline. Thus, examining cognitive correlates of the shared variance between brain markers of pathological aging and everyday functioning may help improve the ecological validity of neuropsychological tests, such that tests associated with the shared variance may be surrogates of or metrics reflecting subtle functional difficulties corresponding to early neurodegenerative changes. In the current study, we used regression analysis to isolate the shared variance between MRI-derived brain variables and an informant report of everyday functioning.

White matter hyperintensities (WMHs), shown as areas of high signal in white matter on T2-weighted magnetic resonance imaging (MRI), are indicators of cerebral small vessel disease and are associated with cognitive decline (Decarli et al., 2001; Brickman et al., 2008; Carmichael et al., 2010; Hu et al., 2021). WMHs have also been associated with a faster rate of everyday functioning decline in older adults without dementia (Puzo et al., 2019; Bangen et al., 2020) and progression from cognitively normal to MCI (DeCarli et al., 2001; Bangen et al., 2018). Hippocampal atrophy is another indicator of neurodegeneration and cognitive decline often observed in Alzheimer’s disease (Barnes et al., 2009; Sabuncu et al., 2011). Studies have shown that smaller hippocampal volumes in cognitively normal individuals are associated with incident MCI (Apostolova and Cummings, 2008; Bangen et al., 2018). Given that changes in these brain structures are often evident long before clinical detection, identifying cognitive correlates of the shared variance between brain volumetric indicators of pathological processes and everyday functioning may have important implications for improving the early detection of neurodegenerative disease.

In addition to identifying neuropsychological markers of pathological everyday functioning, it is important to examine non-cognitive factors that may contribute to daily functioning in order to identify other potentially modifiable risk factors for functional decline. Studies have examined this concept in the context of “functional reserve” or the variance in everyday functioning that is not explained by demographics, neuropsychological performance, or brain volumetrics (Berezuk et al., 2017; Kraal et al., 2021). A recent study examining predictors of functional reserve found that physical function, informant-rated apathy, and informant-rated depression were significantly associated with the residual variance in everyday functioning in a large sample of older adults with normal cognition, MCI, and dementia (Kraal et al., 2021). However, a closer investigation of the way in which correlates of functional reserve may differ as a function of diagnostic status (i.e., cognitively normal vs. MCI) is needed.

In the present study, we aimed (1) to identify the shared variance between brain volumetric indicators of cognitive aging (i.e., WMH and hippocampal volumes) and everyday functioning, (2) to examine demographic, genetic (i.e., APOE ε4 status), and neuropsychological factors associated with the shared variance between brain volumetrics and everyday functioning (i.e., pathological functioning) in individuals with normal cognition and those with MCI, and (3) to identify correlates of the unique or residual variance in everyday functioning (i.e., functional reserve) not accounted for by demographic, neuropsychological, or brain variables. Consistent with these aims, we hypothesized that (1) larger WMH volumes and smaller hippocampal volumes would account for a significant amount of variance in everyday functioning, (2) APOE ε4 carrier status and poorer neuropsychological test performance would be associated with pathological functioning, particularly in the MCI group, and (3) neuropsychiatric symptoms would account for a significant amount of variance in functional reserve.

## Materials and methods

Data used in the current study were derived from the National Alzheimer’s Coordinating Center (NACC) Uniform Data Set (UDS)^1^ up through the September 2020 data freeze and includes data from 14 National Institute on Aging (NIA)-funded Alzheimer’s Disease Research Centers (ADRC). These ADRCs contributed neuropsychological, medical, neuroimaging, and functional data to the NACC-UDS. All ADRCs received approval from their local Institutional Review Boards (IRB) and obtained informed consent from study participants. As determined by the University of Washington Human Subjects Division, the NACC database itself is exempt from IRB review and approval because it does not involve human subjects, as defined by federal and state regulations. In the current study, data from baseline UDS visits conducted between 2005 and 2015 were used in the analyses.

Participants were 600 individuals (168 MCI; 432 cognitively normal) age 55 and older with baseline neuropsychological data, informant-rated everyday functioning, APOE ε4 genotyping, as well as MRI scan data with volumes within 6 months of the baseline UDS visit. Exclusion criteria for the current study were a diagnosis of dementia or other neurological condition (stroke, TBI, seizures, Parkinson’s disease), less than 6 years of formal education, or a Geriatric Depression Scale score of 6 or higher.

### NACC diagnostic classifications

Classification of cognitive status (i.e., cognitively normal vs. MCI) was determined by clinician evaluation or by consensus at each ADRC using all available data including demographics, medical history, medical evaluations, neuropsychological test scores, Clinical Dementia Rating CDR® Dementia Staging Instrument (CDR) score, and informant-rated everyday functioning and emotional functioning (Morris et al., 2006; Beekly et al., 2007; Weintraub et al., 2009). Data included in the current study are from individuals who were classified as either cognitively normal or MCI at the baseline visit.

### Assessment of everyday functioning

The Functional Activities Questionnaire (FAQ) is a 10-item questionnaire (Pfeffer et al., 1982) used to assess everyday functioning including (1) writing checks, paying bills, or balancing a checkbook; (2) assembling tax records, business affairs, or other papers; (3) shopping alone for clothes, household necessities, or groceries; (4) playing a game of skill such as bridge or chess, or working on a hobby; (5) heating water, making a cup of coffee, or turning off the stove; (6) preparing a balanced meal; (7) keeping track of current events; (8) paying attention to and understanding a TV program, book, or magazine; (9) remembering appointments, family occasions, holidays, medications; and (10) traveling out of the neighborhood, driving, or arranging to take public transportation. Each item is rated on a scale from 0 (normal) to 3 (dependent) with a possible range of 0 to 30 with higher total scores indicating greater dependence. If the activity queried was never completed by the participant, it was coded as “not applicable.” For the purposes of the current study, “not applicable” responses were coded as 0 (Teng et al., 2010). Informant ratings of everyday functioning (i.e., FAQ) were completed in interviews with study partners (Weintraub et al., 2009). Mean FAQ scores were used in the analyses.

### Neuropsychological assessment

A standardized battery of neuropsychological tests was administered at each NACC study visit. Details of the NACC-UDS neuropsychological battery have been described previously (Weintraub et al., 2009). The battery included the Wechsler Memory Scale (WMS) Logical Memory, Story A, Immediate and Delayed Recall; Trail Making Test Part A and B; Vegetable Fluency and Animal Fluency; Boston Naming Test (BNT); Digit Span Forward and Backward; and Wechsler Adult Intelligence Scale (WAIS) Digit Symbol. Raw scores were used in all analyses in the present study.

### Genotyping

APOE ε4 status (carrier vs. non-carrier) was derived from the NACC genetic dataset. Participants were classified as APOE ε4 positive if they had at least one APOE ε4 allele.

### MRI brain volumetrics

A subset of Alzheimer’s Disease Centers (ADCs) voluntarily submits MR images to NACC. Imaging data collection and acquisition protocols vary by ADC. The majority of scans were either 1.5 T (*n* = 241) or 3 T (*n* = 273) with the remainder reportedly varying in field strength across images or not reported. MRI manufacturers included GE (*n* = 452), Siemens (*n* = 97), and Philips (*n* = 44) or not reported (*n* = 7). Volumetric MRI quantification was performed by the Imaging of Dementia & Aging (IDeA) Lab (Director: Charles DeCarli, MD; University of California, Davis).^2^ Total WMH volume (in cm^3^) was calculated from T2-weighted fluid-attenuated inversion recovery (FLAIR) images. Details regarding FLAIR image acquisition have been described in a previous publication (Alosco et al., 2018) and processing was similar to that used in ADNI2 (detailed description of processing can be found on the NACC website).^3^ Briefly, the FLAIR image was co-registered to the high-resolution T1-weighted image and inhomogeneity correction was applied to both images (DeCarli et al., 1996). Images were non-linearly transformed into a common template atlas. WMH segmentation was based on a modified Bayesian approach combining image likelihood estimates, spatial priors, and tissue class constraints. WMH prior probability maps were derived based on more than 700 participants using semi-automatic detection of WMH and subsequent manual editing. Likelihood estimates of the native image were calculated *via* histogram segmentation and thresholding. Segmentation was initially performed in standard space resulting in probability likelihood values of WMH at each white matter voxel. These probabilities were then thresholded at >3.5 SD above the mean to create a binary WMH mask (DeCarli et al., 1999). The segmented WMH masks were then back-transformed into native space for volume calculation. Voxels labeled as WMH were summed and multiplied by voxel dimensions to obtain volumes and reported in units of cm^3^. Hippocampal and total cerebral volumes were determined on the T1-weighted image using automated procedures performed by the IDeA lab. The hippocampal segmentation method uses a standard atlas-based diffeomorphic approach (Vercauteren et al., 2007) with minor label refinement modifications. This approach was further modified to include the European Alzheimer’s Disease Consortium (EADC)-Alzheimer’s Disease Neuroimaging Initiative (ADNI) harmonized hippocampal masks to assure standardization across cohorts using the following procedures: (1) Subject image pre-processing with extraction of intracranial cavity, non-uniformity correction, tissue classification; (2) Atlas Registration of all EADC-ADNI hippocampal masks (Frisoni et al., 2013; Boccardi et al., 2015); (3) atlas fusion utilizing Multi-Atlas Label Fusion (Wang et al., 2012); and (4) Intensity-based label refinement. Total hippocampal volume was the sum of right and left hippocampi volumes. Total brain volume was calculated by summing total gray and white matter volumes.

### Neuropsychiatric symptoms

Informant ratings of psychiatric symptoms were obtained *via* structured clinical interviews using the Neuropsychiatric Inventory Questionnaire (NPI-Q; Kaufer et al., 2000). The severity of symptoms ranges from 0 (no symptoms) to 3 (severe symptoms). Single-item ratings of apathy, anxiety and depression were selected for inclusion in the current study based on previous research showing associations with everyday functioning (Hwang et al., 2004; Rog et al., 2014; Kraal et al., 2021).

### Statistical analyses

To establish a pathological functioning score representing the shared variance between brain volumetric indicators and everyday functioning, using a hierarchical regression analysis, mean FAQ scores (log-transformed to improve distribution normality due to positive skew) were regressed on total hippocampal volume and total white matter hyperintensity volume (log-transformed to improve distribution normality due to positive skew), adjusting for log-transformed total intracranial volume (ICV). Both the standardized predicted (i.e., pathological functioning variable) and standardized residual (i.e., functional reserve variable) values were used as dependent variables in subsequent analyses. The functional reserve variable was reverse signed so that higher values indicate greater functional reserve.

To identify cognitive correlates of pathological functioning, hierarchical regression analyses were conducted with the pathological functioning value regressed on age, sex, race/ethnicity, and APOE ε4 status (carrier vs. non-carrier) in block 1, and neuropsychological test scores in block 2. Separate regression analyses were used to examine cognitive correlates of pathological functioning in the cognitively normal group and those classified as MCI.

To identify neuropsychiatric correlates of functional reserve, the residual variance value was regressed on NPI-Q apathy, depression, and anxiety scores (in block 3), adjusting for demographics and APOE ε4 status (in block 1), and neuropsychological test variables (in block 2). These analyses were stratified by cognitive status (cognitively normal, MCI). Possible multicollinearity effects were examined and determined to be within an acceptable range (i.e., all VIFs <4).

## Results

### Participant characteristics and descriptive statistics

Sample characteristics are shown in Table 1. As expected, the MCI group was older, more likely to be APOE ε4 positive, and had lower neuropsychological test scores, higher mean FAQ scores, smaller hippocampal volumes, and greater WMH volume compared to cognitively normal participants. The MCI group also had higher informant-rated depression, anxiety, and apathy severity.

**Table 1:** Participant characteristics.

	Total sample (*N* = 600)		Cognitively normal (*n* = 432)		MCI (*n* = 168)			Effect size	
	*M*	SD	*M*	SD	*M*	SD	*F* or *X*^2^	Hedge’s *g* or phi	*p*
Demographics									
Age	71.32	8.67	69.75	8.63	75.36	7.40	55.02	0.674	<0.001
Sex (% female)	58.5		62.5		48.2		10.16	0.130	0.002
Education	15.50	3.10	15.63	3.04	15.15	3.26	2.93	0.155	0.087
Race/ethnicity (% NHW)	91.2		90.5		92.9		0.828	0.037	0.425
Genetics									
APOE ε4 (% positive)	36.7		32.9		46.4		9.57	0.126	0.002
Neuropsychological									
LM I – Immediate Recall	11.46	4.53	12.93	3.94	7.68	3.68	222.24	1.354	<0.001
LM II – Delayed Recall	9.81	5.11	11.69	4.23	4.96	3.81	322.94	1.632	<0.001
Digit Span Forward	8.29	2.03	8.38	2.07	8.07	1.91	2.79	0.152	0.095
Digit Span Backward	6.47	2.21	6.74	2.23	5.76	2.01	24.51	0.450	<0.001
Animal Fluency	19.50	6.09	21.09	5.77	15.42	4.87	127.43	1.025	<0.001
Vegetable Fluency	13.69	4.69	15.02	4.40	10.27	3.53	155.76	1.133	<0.001
Digit Symbol	45.66	12.81	49.12	11.71	36.76	11.15	138.23	1.068	<0.001
Boston Naming Test	26.59	4.06	27.67	2.86	23.82	5.22	132.88	1.047	<0.001
Trails A	36.22	18.83	32.05	13.70	46.95	25.05	86.44	0.844	<0.001
Trails B	102.57	62.31	83.94	42.78	150.49	77.42	179.03	1.215	<0.001
Everyday functioning									
FAQ	0.15	0.36	0.05	0.20	0.42	0.50	168.64	1.179	<0.001
Brain volumetrics									
Total Brain volume	0.71	0.16	0.72	0.16	0.69	0.16	3.78	0.344	0.052
Total WMH volume	1.11	2.16	0.82	2.18	1.83	1.92	28.17	0.482	<0.001
Hippocampal volume	6.26	0.81	6.44	0.71	5.81	0.87	82.63	0.764	<0.001
Neuropsychiatric									
NPI-Q Depression	0.20	0.52	0.14	0.45	0.34	0.63	19.02	0.397	<0.001
NPI-Q Anxiety	0.17	0.48	0.09	0.35	0.34	0.68	34.97	0.538	<0.001
NPI-Q Apathy	0.11	0.38	0.05	0.27	0.23	0.55	29.63	0.495	<0.001

Effect sizes are in terms of phi for categorical variables and Hedge’s g for continuous variables.

### Brain volumetric correlates of everyday functioning

In the hierarchical multiple regression analysis, ICV was entered in the first block and WMH volume and hippocampal volume variables were entered into the second block. The results indicated that the first block was not statistically significant [*R*^2^ = 0.000, *F*(1, 598) = 0.078, *p* = 0.781]. Results of the second block indicated that the model was statistically significant [Δ*R*^2^ = 0.089, Δ*F*(2, 596) = 28.938, *p* < 0.001]. Greater total WMH volumes (*B* = 0.024, SE = 0.006, *β* = 0.229, *t* = 4.036, *p* < 0.001) and smaller hippocampal volumes (*B* = −0.056, SE = 0.012, *β* = −0.200, *t* = −4.783, *p* < 0.001) were significantly associated with higher FAQ scores. The standardized predicted variance value was used as the pathological functioning score, with higher values indicating greater brain-related difficulties with everyday functioning. The residual variance, representing everyday functioning unrelated to normalized total brain volume, hippocampal volume, and WMH volume, was used in subsequent functional reserve analyses.

Given that only 460 of the 600 participants had complete data for all 10 FAQ items, an exploratory regression analysis was conducted that included only those with complete FAQ data to examine whether the results differed if those with missing data were excluded. Consistent with the analysis of the whole sample, block 1 was non-significant [*R*^2^ = 0.000, *F*(1, 458) = 0.212, *p* = 0.645] and block 2 was statistically significant [Δ*R*^2^ = 0.083, Δ*F*(2, 456) = 20.697, *p* < 0.001] with WMH volumes [*B* = 0.015, SE = 0.006, *β* = 0.172, *t* = 2.632, *p* = 0.009] and hippocampal volumes (*B* = −0.054, SE = 0.012, *β* = −0.223, *t* = −4.637, *p* < 0.001) associated with mean FAQ scores. Therefore, data from the entire sample (*n* = 600) was used in the analyses.

### Neuropsychological correlates of pathological functioning

In the hierarchical regression analysis for the MCI group (see Table 2), demographic characteristics and APOE ε4 status were entered into the first block and neuropsychological variables were entered into the second block. Results of the model in the first block were statistically significant [*R*^2^ = 0.212, *F*(5, 162) = 8.729, *p* < 0.001]. Older age, female sex, and APOE ε4 positivity were associated with a higher pathological functioning score. The model in the second block was statistically significant [Δ*R*^2^ = 0.154, Δ*F*(10, 152) = 3.680, *p* < 0.001], and performances on delayed recall (LMII) and vegetable fluency were negatively, and digit symbol substitution test positively associated with the pathological functioning score. A trend-level association was observed for BNT (*p* = 0.050).

**Table 2:** Hierarchical regression model of cognitive correlates of pathological functioning in MCI group.

Model					*t*	*p*
		*B*	SE	*β*		
1	(Constant)	−3.423	0.978		−3.499	<0.001
	Age	0.044	0.009	0.346	4.755	<0.001
	Sex	0.554	0.138	0.297	4.023	<0.001
	Education	−0.021	0.021	−0.074	−1.002	0.318
	Race/ethnicity	0.077	0.259	0.021	0.297	0.766
	E4 status	0.382	0.135	0.204	2.827	0.005
	*R*^2^ = 0.233, *F*(5, 162) = 9.845, *p* < 0.001					
2	(Constant)	−2.317	1.115		−2.079	0.039
	Age	0.041	0.009	0.323	4.425	<0.001
	Sex	0.632	0.145	0.339	4.354	<0.001
	Education	−0.026	0.022	−0.090	−1.168	0.245
	Race/ethnicity	−0.018	0.254	−0.005	−0.072	0.943
	E4 status	0.250	0.132	0.134	1.901	0.059
	LM I - Immediate Recall	0.037	0.028	0.145	1.307	0.193
	LM II - Delayed Recall	−0.068	0.027	−0.275	−2.474	0.014
	Digit Span Forward	−0.056	0.039	−0.115	−1.439	0.152
	Digit Span Backward	0.036	0.039	0.078	0.929	0.354
	Animal Fluency	−0.002	0.016	−0.011	−0.127	0.899
	Vegetable Fluency	−0.052	0.022	−0.198	−2.390	0.018
	Digit Symbol	0.016	0.008	0.193	2.060	0.041
	Boston Naming Test	−0.028	0.014	−0.157	−1.962	0.052
	Trails A	0.006	0.003	0.158	1.818	0.071
	Trails B	−0.001	0.001	−0.059	−0.658	0.511
	Δ*R*^2^ = 0.146, Δ*F*(10, 152) = 3.561, *p* < 0.001					

*N* = 168.

In the cognitively normal group (see Table 3), results of the hierarchical regression revealed that the first block was significant [*R*^2^ = 0.379, *F*(5, 426) = 51.892, *p* < 0.001] and that older age, female sex, less education, and non-NHW race/ethnicity was associated with greater pathological functional impairment. The model in the second block was statistically significant [Δ*R*^2^ = 0.032, Δ*F*(10, 416) = 2.274, *p* = 0.013], with only Digit Span Backwards as a significant predictor.

**Table 3:** Hierarchical regression model of cognitive correlates of pathological functioning in cognitively normal group.

Model					*t*	*p*
		*B*	SE	*β*		
1	(Constant)	−5.027	0.449		−11.185	<0.001
	Age	0.060	0.004	0.570	14.812	<0.001
	Sex	0.442	0.072	0.234	6.137	<0.001
	Education	−0.031	0.012	−0.104	−2.577	0.010
	Race/ethnicity	0.275	0.125	0.088	2.209	0.028
	E4 status	0.122	0.074	0.063	1.656	0.099
	*R*^2^ = 0.399, *F*(5, 426) = 56.553, *p* < 0.001					
2	(Constant)	−3.244	0.723		−4.485	<0.001
	Age	0.050	0.005	0.474	10.092	<0.001
	Sex	0.458	0.080	0.243	5.762	<0.001
	Education	−0.007	0.013	−0.025	−0.567	0.571
	Race/ethnicity	0.115	0.129	0.037	0.892	0.373
	E4 status	0.078	0.073	0.040	1.068	0.286
	LM I - Immediate Recall	0.016	0.016	0.070	0.991	0.322
	LM II - Delayed Recall	−0.023	0.015	−0.108	−1.540	0.124
	Digit Span Forward	−0.005	0.020	−0.011	−0.227	0.821
	Digit Span Backward	−0.047	0.019	−0.115	−2.434	0.015
	Animal Fluency	−0.006	0.008	−0.039	−0.755	0.451
	Vegetable Fluency	−0.004	0.010	−0.017	−0.347	0.729
	Digit Symbol	−0.004	0.005	−0.046	−0.770	0.442
	Boston Naming Test	−0.019	0.015	−0.059	−1.260	0.208
	Trails A	−0.001	0.003	−0.011	−0.223	0.824
	Trails B	0.000	0.001	0.019	0.319	0.750
	Δ*R*^2^ = 0.033, Δ*F*(10, 416) = 2.419, *p* = 0.008					

*N* = 432.

### Neuropsychiatric correlates of functional reserve

The residual variance from the regression analysis with brain volumetrics predicting FAQ scores was used as the dependent variable in the functional reserve analyses. As with the previous analyses, demographics, APOE ε4 status, and neuropsychological variables were entered into the first blocks (block 1 and block 2) and then NPI-Q depression, anxiety, and apathy were entered into the final block (block 3) to examine the unique association of emotional factors in everyday functioning.

In the MCI group (*n* = 167), block 1 was significant [*R*^2^ = 0.085, *F*(5, 161) = 3.009, *p* = 0.013], with male sex as the only statistically significant variable [*B* = 0.709, SE = 0.223, *β* = 0.256, *t* = 3.171, *p* = 0.002]. Block 2, with neuropsychological variables, was not statistically significant [Δ*R*^2^ = 0.091, Δ*F*(10, 151) = 1.662, *p* = 0.095]. Block 3 was statistically significant [Δ*R*^2^ = 0.118, Δ*F*(3, 148) = 8.230, *p* < 0.001], and only anxiety severity was associated with the residual variance score (*B* = −0.692, SE = 0.164, *β* = −0.342, *t* = −4.220, *p* < 0.001), such that greater anxiety was associated with lower functional reserve.

In the cognitively normal group, block 1 was significant [*R*^2^ = 0.048, *F*(5, 426) = 4.250, *p* < 0.001], and older age (*B* = 0.010, SE = 0.004, *β* = 0.142, *t* = 2.930, *p* = 0.004), male sex (*B* = 0.185, SE = 0.062, *β* = 0.143, *t* = 2.986, *p* = 0.003), and APOE ε4 negativity (*B* = −0.129, SE = 0.064, *β* = −0.097, *t* = −2.019, *p* = 0.044) were associated with the residual variance score. Neuropsychological variables did not account for a significant amount of variance in block 2 [Δ*R*^2^ = 0.036, Δ*F*(10, 416) = 1.642, *p* = 0.092]. Results in block 3 were significant [Δ*R*^2^ = 0.087, Δ*F*(3, 413) = 14.412, *p* < 0.001], with greater apathy severity (*B* = −0.462, SE = 0.106, *β* = −0.207, *t* = −4.353, *p* < 0.001) and greater depression severity (*B* = −0.208, SE = 0.068, *β* = −0.151, *t* = −3.033, *p* = 0.003) associated with lower functional reserve.

## Discussion

We found that pathological everyday functioning, defined as the shared variance between informant-rated FAQ scores and MRI brain variables (i.e., WMH and hippocampal volumes), was differentially associated with cognition as a function of cognitive status (MCI vs. cognitively normal). In the MCI group, cognitive variables accounted for 15.5% of the total variance in pathological functioning compared to 3.2% in the cognitively normal group. These findings suggest that subtle brain-related everyday functional difficulties are evident in individuals without dementia and correspond to the level of cognitive functioning observed on neuropsychological measures. Previous research has shown that mild functional impairments are present in MCI (Burton et al., 2009; Bangen et al., 2010; Teng et al., 2010) as well as cognitively normal individuals (Farias et al., 2017). Our results support and expand these findings by demonstrating aspects of cognitive functioning in MCI that are associated with everyday functioning that is uniquely related to MRI brain variables indicative of pathological processes (e.g., atrophy, white matter disease). This approach provides a clearer link between cognition and everyday functioning that is not affected by potentially confounding factors such as peripherally-mediated physical functioning and/or emotional distress.

Our secondary aim was to examine neuropsychiatric correlates of functional reserve, the unique variance in everyday functioning not accounted for by demographics, cognition, or brain volumetrics. Consistent with prior research using samples of adults with normal cognition, MCI, and dementia (Kraal et al., 2021), we found that greater neuropsychiatric symptomatology was associated with lower functional reserve. We extended prior work by stratifying our sample by cognitive status (cognitively normal, MCI) and found that in the MCI group, only anxiety severity, but not depression or apathy, was associated with lower functional reserve in the multivariable model. The lack of association of depression and apathy with functional reserve in the MCI group was unexpected, particularly because these symptoms are prevalent in MCI and previous studies have shown an association with everyday functioning (Zahodne and Tremont, 2013; Rog et al., 2014) in MCI. However, these prior studies did not examine the extent to which neuropsychiatric factors are associated with functional reserve, which may account for the discrepant findings.

Given the evidence that both WMH and hippocampal volume predict incident AD in older adults without dementia (Brickman et al., 2015), and that acceleration of WMH accumulation precedes clinically detected cognitive impairment (Silbert et al., 2008, 2009, 2012), we were interested in examining whether these variables were associated with subtle functional difficulties in older adults without dementia. Consistent with previous studies, we found that greater WMH and smaller hippocampal volumes accounted for a significant amount of variance in informant-rated everyday functioning among older adults without dementia (DeCarli et al., 2001; Apostolova and Cummings, 2008; Bangen et al., 2018). By combining these variables into a single metric (i.e., pathological functioning), we could be inclusive of common early etiologic indicators of cognitive decline, specifically, cerebral small vessel disease and neurodegeneration consistent with AD pathogenesis. This approach is advantageous because it restricts informant–rated functional ability into a variable that fully overlaps with MRI indicators of brain pathology and, thus, is likely to be more sensitive to subtle cognitive changes.

In the MCI group, the pattern of association between cognitive variables and pathological functioning was consistent with an AD process such that lower verbal memory and semantic fluency scores predicted pathological everyday functioning in the cross-sectional regression analysis. This finding supports the ecological validity of these measures in detecting early IADL changes as they were significantly associated with brain-related aspects of everyday functioning. In contrast, a measure of processing speed (i.e., digit symbol) was associated with pathological functioning, although it was in the opposite direction than expected such that faster processing speed was associated with greater pathological functioning. A similar trend (*p* = 0.064) was observed for Trails A performance. The reason for this counterintuitive direction is unclear and requires further investigation. Follow-up partial correlations used to examine this unexpected result revealed that the association between processing speed and pathological functioning was not statistically significant (see Appendix). The lack of significant association between executive functioning (as measured by Trails B performance) and everyday functioning in the current study contrasts with a meta-analytic study (McAlister et al., 2016) showing Trails B to be the strongest cognitive predictor of functional status in MCI. Although WMH volumes have been linked to poorer executive functioning in older adults (e.g., Boutzoukas et al., 2021), in the current study we did not examine other brain variables associated with executive functioning such as frontal lobe structures, which may account for the null association we found between Trails B and pathological everyday functioning. It is also likely that this difference is due to the large representation of amnestic MCI (84.5%) subtype in the composition of the sample used in our study.

Prior work has shown that early alterations in attentional control are sensitive to AD-related biomarkers in cognitively healthy individuals (Aschenbrenner et al., 2015, 2020; McKay et al., 2022). Consistent with previous research, in the current study working memory (Digit Span Backward) was the only cognitive predictor of pathological functioning in the cognitively normal group. This finding suggests that subtle cognitive changes in cognitively normal individuals may correspond to very early mild brain-related functional changes. However, given the relatively small effect, further research is needed to verify the reliability of this association.

Although cognitive variables accounted for a significant portion of pathological functioning variance across diagnostic groups, the contribution of demographic characteristics and APOE ε4 carrier status to the regression models were notable. In the initial models, these variables accounted for approximately 21% and 38% of the variance in pathological functioning in MCI and cognitively normal individuals, respectively. In the MCI group, older age, female sex, and APOE ε4 status remained significant correlates of pathological functioning after the inclusion of neuropsychological variables in the model. Among cognitively normal participants, older age, female sex, less education, and self-identifying as not non-Hispanic white were significant correlates in the initial regression model, but only age and sex remained significant after including cognitive variables. These findings raise the possibility that traditional neuropsychological tests are not capturing the full spectrum of age-related cognitive changes involved in complex activities of daily living. Although the measures used in the current study have been well-validated and spanned multiple cognitive domains, some aspects of cognition relevant to cognitive aging were not examined, notably, several aspects of memory such as prospective memory, temporal order memory, and visual memory. Indeed, prior work has shown that performance on novel memory paradigms is associated with self-reported, informant-reported and directly observed measures of everyday functioning (Fellows and Schmitter-Edgecombe, 2018). It is also possible that physical capacity may mediate or moderate the association between age and pathological functioning, independent from cognition. Studies have shown that WMHs (Willey et al., 2013), and smaller medial temporal regions (Rosano et al., 2012; Ezzati et al., 2015) are associated with slowed gait speed. Reduced mobility has been shown to mediate the association between age and the ability to perform daily activities that require physical capacity (Fellows and Schmitter-Edgecombe, 2019). These studies suggest pathways between age-related brain changes and mobility that may influence everyday functioning independently from cognition. Yet, given the shared etiology of mobility and cognitive changes *via* global WMH and medial temporal atrophy, these factors may exert an additive effect on everyday functioning.

The NACC data used in this study include a large well-characterized group of participants with neuropsychological and neuroimaging data. However, the participants are generally highly educated and mostly non-Hispanic white, which limits the ability to generalize findings to more diverse groups that represent nearly 40% of the U.S. adult population (U.S. Census, 2020). Although the NACC dataset overall has greater racial/ethnic diversity than other large prospective cohort studies (Kiselica and Benge, 2021), restricting inclusion in the current study to only individuals who had neuropsychological, MRI, and everyday functioning data resulted in a sample that was >90% non-Hispanic white. Further, Gleason et al. (2019) demonstrated that NACC study enrollment factors, such as referral source (i.e., Black participants being over-sampled in community-based recruitment and under-sampled in clinic-based recruitment), introduce further racial/ethnic bias into an already under-representative sample of Black participants. In the current study, race/ethnicity was significantly associated with pathological functioning in the cognitively normal group but the correlation was attenuated to a non-significant level after the inclusion of cognitive variables in the regression model. This finding may suggest a possible interactive effect between race/ethnicity and cognition on pathological functioning, however, due to the small sample size and bias in sampling, further examination of this effect could not be examined. Greater diversity in large research cohorts is needed to better understand the complex factors contributing to racial and ethnic differences in cognitive aging.

The cross-sectional and observational design of the analyses used in the current study precludes the interpretation of causality. Additionally, by design, participants included in this study had only minimal informant-rated functional difficulties and minimal depressive symptoms. Future research examining the full spectrum of cognitive aging without exclusion of individuals with more severe emotional distress is needed to further elucidate the continuum of pathological functioning and functional reserve.

In summary, we found that lower hippocampal volumes and greater total WMH volumes are independently associated with increased functional difficulties in adults without dementia. Our findings indicate that subtle brain-related everyday functioning difficulties are evident in MCI and correspond with the preclinical Alzheimer’s disease cognitive phenotype (lower verbal memory and category fluency) which highlights the importance of neuropsychological assessment in the early detection of pathological cognitive aging. We also found that even relatively low levels of informant-rated neuropsychiatric symptoms were associated with lower functional reserve and may be targets for early therapeutic intervention.

## Data availability statement

Publicly available datasets were analyzed in this study. This data can be found at: https://naccdata.org/.

## Author contributions

RF designed the study, analyzed and interpreted the data, and drafted the manuscript. KB, LG, LD-W, and MB contributed critical intellectual content to manuscript revisions. All authors contributed to the article and approved the submitted version.

## Funding

This work was supported by Grants R01 AG049810 and R01 AG063782. The NACC database is funded by NIA/NIH Grant U24 AG072122. NACC data are contributed by the NIA-funded ADCs: P50 AG005131 (PI James Brewer, MD, PhD), P50 AG005133 (PI Oscar Lopez, MD), P50 AG005134 (PI Bradley Hyman, MD, PhD), P50 AG005136 (PI Thomas Grabowski, MD), P50 AG005138 (PI Mary Sano, PhD), P50 AG005142 (PI Helena Chui, MD), P50 AG005146 (PI Marilyn Albert, PhD), P50 AG005681 (PI John Morris, MD), P30 AG008017 (PI Jeffrey Kaye, MD), P30 AG008051 (PI Thomas Wisniewski, MD), P50 AG008702 (PI Scott Small, MD), P30 AG010124 (PI John Trojanowski, MD, PhD), P30 AG010129 (PI Charles DeCarli, MD), P30 AG010133 (PI Andrew Saykin, PsyD), P30 AG010161 (PI David Bennett, MD), P30 AG012300 (PI Roger Rosenberg, MD), P30 AG013846 (PI Neil Kowall, MD), P30 AG013854 (PI Robert Vassar, PhD), P50 AG016573 (PI Frank LaFerla, PhD), P50 AG016574 (PI Ronald Petersen, MD, PhD), P30 AG019610 (PI Eric Reiman, MD), P50 AG023501 (PI Bruce Miller, MD), P50 AG025688 (PI Allan Levey, MD, PhD), P30 AG028383 (PI Linda Van Eldik, PhD), P50 AG033514 (PI Sanjay Asthana, MD, FRCP), P30 AG035982 (PI Russell Swerdlow, MD), P50 AG047266 (PI Todd Golde, MD, PhD), P50 AG047270 (PI Stephen Strittmatter, MD, PhD), P50 AG047366 (PI Victor Henderson, MD, MS), P30 AG049638 (PI Suzanne Craft, PhD), P30 AG053760 (PI Henry Paulson, MD, PhD), P30 AG066546 (PI Sudha Seshadri, MD), P20 AG068024 (PI Erik Roberson, MD, PhD), P20 AG068053 (PI Marwan Sabbagh, MD), P20 AG068077 (PI Gary Rosenberg, MD), P20 AG068082 (PI Angela Jefferson, PhD), P30 AG072958 (PI Heather Whitson, MD), and P30 AG072959 (PI James Leverenz, MD).

## Conflict of interest

The authors declare that the research was conducted in the absence of any commercial or financial relationships that could be construed as a potential conflict of interest.

## Publisher’s note

All claims expressed in this article are solely those of the authors and do not necessarily represent those of their affiliated organizations, or those of the publisher, the editors and the reviewers. Any product that may be evaluated in this article, or claim that may be made by its manufacturer, is not guaranteed or endorsed by the publisher.

## Figures and Tables

**Table:** 

	*r*_partial_	*p*
LM I – Immediate Recall	−0.178	0.023
LM II – Delayed Recall	−0.261	<0.001
Digit Span Forward	−0.042	0.597
Digit Span Backward	0.052	0.509
Animal Fluency	−0.164	0.036
Vegetable Fluency	−0.243	0.002
Digit Symbol	0.047	0.548
Boston Naming Test	−0.257	<0.001
Trails A	0.133	0.090
Trails B	−0.034	0.664

Partial correlations are adjusted for age, sex, race/ethnicity, education, and APOE ε4 carrier status.

## Footnotes

^1^ http://www.alz.washington.edu

^2^ http://idealab.ucdavis.edu/

^3^ https://files.alz.washington.edu/documentation/adni-proto.pdf

## Appendix

Partial correlations between pathological functioning and cognitive variables in MCI group.
